# Characterization of Putative Erythroid Regulators of Hepcidin in Mouse Models of Anemia

**DOI:** 10.1371/journal.pone.0171054

**Published:** 2017-01-30

**Authors:** Cornel S. G. Mirciov, Sarah J. Wilkins, Linda A. Dunn, Gregory J. Anderson, David M. Frazer

**Affiliations:** 1 Iron Metabolism Laboratory, QIMR Berghofer Medical Research Institute, Herston, Queensland, Australia; 2 School of Medicine, The University of Queensland, St Lucia, Queensland, Australia; 3 School of Chemistry and Molecular Bioscience, The University of Queensland, St Lucia, Queensland, Australia; Lady Davis Institute for Medical Research, CANADA

## Abstract

Iron is crucial for many biological functions, but quantitatively the most important use of iron is in the production of hemoglobin in red blood cell precursors. The amount of iron in the plasma, and hence its availability for hemoglobin synthesis, is determined by the liver-derived iron regulatory hormone hepcidin. When the iron supply to erythroid precursors is limited, as often occurs during stimulated erythropoiesis, these cells produce signals to inhibit hepatic hepcidin production, thereby increasing the amount of iron that enters the plasma. How stimulated erythropoiesis suppresses hepcidin production is incompletely understood, but erythroferrone, Gdf15 and Twsg1 have emerged as candidate regulatory molecules. To further examine the relationship between erythropoiesis and the candidate erythroid regulators, we have studied five mouse models of anemia, including two models of β-thalassemia (*Hbb*^*th3/+*^ and *RBC14*), the hemoglobin deficit mouse (*hbd*), dietary iron deficient mice and mice treated with phenylhydrazine to induce acute hemolysis. Hematological parameters, iron status and the expression of *Erfe* (the gene encoding erythroferrone), *Gdf15* and *Twsg1* in the bone marrow and spleen were examined. *Erfe* expression was the most consistently upregulated of the candidate erythroid regulators in all of the mouse models examined. Gene expression was particularly high in the bone marrow and spleen of iron deficient animals, making erythroferrone an ideal candidate erythroid regulator, as its influence is strongest when iron supply to developing erythroid cells is limited. *Gdf15* expression was also upregulated in most of the anemia models studied although the magnitude of the increase was generally less than that of *Erfe*. In contrast, very little regulation of *Twsg1* was observed. These results support the prevailing hypothesis that erythroferrone is a promising erythroid regulator and demonstrate that *Erfe* expression is stimulated most strongly when the iron supply to developing erythroid cells is compromised.

## Introduction

The peptide hormone hepcidin is often referred to as the master regulator of iron homeostasis because it regulates both the amount of iron absorbed from the diet and the release of iron from intracellular storage sites (predominantly hepatocytes and macrophages) [1]. Hepcidin achieves this by binding to and triggering the degradation of the cell surface iron export protein ferroportin, thereby inhibiting cellular iron release [2]. Encoded by the *HAMP* gene, hepcidin is produced predominantly by hepatocytes, which secrete the active peptide into the circulation [3, 4]. The major regulators of hepatic *HAMP* expression are body iron stores, often referred to as the stores regulator, and the iron demands of developing erythroid cells, commonly called the erythroid regulator [1]. The molecular basis of the stores regulator is relatively well understood. An increase in iron levels within the body stimulates the production of bone morphogenetic protein 6 (BMP6) by non-parenchymal cells in the liver [5]. BMP6 binds to the BMP receptor complex on the surface of hepatocytes, resulting in activation of the SMAD pathway, which, in turn, directly stimulates the *HAMP* promoter and hepcidin production [6]. This pathway allows the stores regulator to adjust circulating hepcidin levels to ensure that body iron levels.

In contrast, the molecular basis of the erythroid regulator is less clear. Its purpose is to ensure that the iron demands of developing erythroid cells are met [7]. If iron supply is limited, the erythroid regulator inhibits the production of hepcidin by hepatocytes, elevating both dietary iron absorption and storage iron release, thereby providing the extra iron necessary for red blood cell production [1]. Activation of the erythroid regulator is evident in pathological conditions such as β-thalassemia that are characterized by ineffective erythropoiesis. This spectrum of disorders, which can range in severity from asymptomatic to life threatening, is caused by defective β-globin production [8]. This leads to premature red blood cell destruction and anemia. If the iron demand associated with this ineffective erythropoiesis exceeds the iron supply to the marrow, the erythroid regulator is triggered and hepcidin production is inhibited, causing an increase in dietary iron absorption and tissue iron loading.

In order to relay the iron requirements of developing erythroid cells in the bone marrow (and the spleen during stress erythropoiesis) to hepatocytes expressing hepcidin, the mediator of the erythroid regulator must circulate in the bloodstream. Previously proposed signalling molecules include growth differentiation factor 15 (GDF15), a member of the transforming growth factor β superfamily of cytokines [9], and the BMP agonist/antagonist twisted gastrulation BMP signalling modulator 1 (TWSG1) [9, 10]. Both molecules were first identified as genes upregulated in an *in vitro* model of human erythroblast differentiation, with GDF15 highly expressed during late differentiation and TWSG1 more prominent during early erythroid development [9]. The link with iron homeostasis came when treatment of primary human hepatocytes and the human hepatoma cell line HuH7 with GDF15 or TWSG1 inhibited the expression of *HAMP* [9, 10]. The genes encoding both molecules were also upregulated in the bone marrow and spleen of mouse models of β-thalassemia [9, 10]. However, a subsequent study showed that *Gdf15* knockout mice were able to decrease *Hamp1* expression similarly to wild-type mice following blood loss, indicating that, at least in mice, *Gdf15* is not essential for hepcidin inhibition following phlebotomy [11]. This study does not exclude a role for GDF15 as an erythroid regular, but it makes it unlikely it is the sole regulator. Further studies involving the overexpression of *Gdf15* are required to determine whether it does play any role in hepcidin regulation *in vivo*. No such study has been carried out in *Twsg1* knockout mice. *Twsg1* mRNA expression in the bone marrow and spleen does not change in mice after blood loss or erythropoietin treatment despite significant decreases in *Hamp1* expression [12]. Again, these studies were not conclusive as serum Twsg1 levels were not measured. Although these studies do not discount a role for GDF15 or TWSG1 as erythroid regulators, they do indicate that other molecules are also likely to be involved.

More recently, erythroferrone, a member of the tumour necrosis factor superfamily of cytokines, was proposed as a candidate erythroid regulator [13]. Although initially characterised as a cytokine secreted by muscle tissue and involved in the regulation of lipid metabolism and cellular autophagy [14–16], Kautz et al. showed that *Erfe* (the gene encoding erythroferrone in mice) expression was increased in the bone marrow and spleen of mice following blood loss or erythropoietin administration, stimuli that increase red blood cell production [13]. Furthermore, following phlebotomy, *Erfe* knockout mice failed to decrease hepatic *Hamp1* expression to the same level as wild-type controls [13], providing convincing *in vivo* evidence that erythroferrone is an erythroid regulator of hepcidin. In addition, recombinant erythroferrone injection or lentivirus-mediated erythroferrone expression in mice decreased hepatic *Hamp1* expression as well as serum hepcidin levels [13]. However, although β-thalassemic mice lacking erythroferrone had *Hamp1* expression levels comparable to wild-type mice, the liver iron concentration in these animals was still significantly higher than wild-type values, implying that additional erythroid factors are involved [13]. It is likely, therefore, that multiple erythroid regulators exist and act to regulate hepcidin production in response to stimulated erythropoiesis.

While the expression of *Erfe*, *Gdf15* and *Twsg1* has been evaluated in *Hbb*^*th3/+*^ mice, a commonly used mouse model of β-thalassemia [13], their expression in other conditions associated with increased erythropoietic drive has not been thoroughly examined. In the current study, we have investigated the expression of the three candidate erythroid regulators in various mouse models of anemia. While the genes encoding erythroferrone and Gdf15 were upregulated in the bone marrow and spleen in most of the models examined, the highest levels were seen with iron deficiency. In contrast, *Twsg1* expression showed little change in any of the models.

## Materials and Methods

### Animal models

This study utilized five well characterised models of anemia, including three genetic anemias, as well as dietary iron deficiency and hemolysis in wild-type mice. For all studies the mice were on a C57BL/6J background and only male mice were used. *Hbb*^*th3/+*^ mice are a widely used model of β-thalassemia intermedia and exhibit both anemia and iron loading [17]. The recently described *RBC14* mouse is also a model of β-thalassemia, with a mutation matching that found in a case of human β-thalassemia [18], however, the phenotype of this strain is much less severe than that of *Hbb*^*th3/+*^ mice [19]. *Hbd* mice have a deletion in the *Sec15l1* gene, which reduces transferrin-bound iron delivery to developing erythrocytes, causing microcytic, hypochromic anemia [20]. These three mouse strains were weaned onto a standard rodent pellet diet (120 mg/kg iron, Norco Stockfeed, Lismore, Australia) at 21 days of age and euthanized at 3.5 weeks of age for tissue collection. This age was chosen to ensure that the effect of the erythroid regulator was at its strongest, as previous studies using *Hbb*^*th3/+*^ mice show that *Hamp1* expression increases rapidly to wild-type levels as the animals age [21]. Wild-type littermates were used as controls for all strains. We also examined wild-type mice with iron deficiency anemia (IDA) and those in which acute hemolysis had been induced using phenylhydrazine (PHZ). To induce iron deficiency anemia, C57BL/6J dams with litters (Animal Resources Centre, Perth, Australia) were switched to a very low iron diet (1mg Fe/kg [22], SF01-017, Specialty Feeds, Glen Forrest, Australia) when the pups were 2 weeks of age. Male pups were weaned onto the same iron deficient diet at 3 weeks of age and maintained on this diet for a further 5 weeks. Mice maintained under the same conditions on a control diet (68 mg Fe/kg, AIN-93G, Specialty Feeds) were used for comparison to these animals. To induce acute hemolysis, 6 week old C57BL/6J mice (Animal Resources Centre) were injected intraperitoneally with 100mg/kg of PHZ and examined 4 days after injection [23]. Untreated C57BL/6J mice were used as controls.

Prior to euthanasia, all animals were anesthetized with 200mg/kg ketamine and 10mg/kg xylazine and blood taken by cardiac puncture for hematological and serum analysis. Liver, spleen and bone marrow were snap frozen in liquid nitrogen for subsequent analysis. All experiments were carried out in strict accordance with the recommendations in the Australian Code for the Care and Use of Animals for Scientific Purposes, 8^th^ Edition, 2013. Protocols were approved by the QIMR Berghofer Animal Ethics Committee (approval number A0912-609M). All efforts were made to minimise animal suffering.

### Hematological parameters, erythropoietin levels and iron status

Hematological parameters were measured using a Sysmex XE-5000 automated hematology analyser (Roche Diagnostics, Castle Hill, Australia) at Pathology Queensland, Royal Brisbane and Women’s Hospital (Brisbane, Australia). Mouse erythropoietin was determined using a commercial ELISA kit (MEP00B, In Vitro Technologies, Noble Park, Australia) according to the manufacturer’s instructions. Total serum iron concentration and transferrin saturation were measured using the Iron/TIBC Reagent Set (I7504, Pointe Scientific, MI). A colorimetric assay was used to measure the concentration of non-heme iron in the liver as previously described [24].

### RNA extraction and gene expression analysis

TRIzol reagent (Thermo Fisher Scientific, Scoresby, Australia) was used to extract total RNA from each sample according to the manufacturer’s instructions. Complementary DNA (cDNA) was synthesized using SuperScript III reverse transcriptase (Thermo Fisher Scientific) and an oligo (dT) primer according the manufacturer’s instructions. Hepatic cDNA was synthesised using 500ng of RNA. Bone marrow and spleen cDNA were synthesised using 2000ng of RNA. Real time quantitative polymerase chain reaction (qPCR) was performed on a Light Cycler 480 (Roche Diagnostics). Each sample was analysed in triplicate and gene expression was calculated from the C_t_ value using the standard curve method. We have normalized gene expression to the housekeeper gene *ribosomal protein L13A (Rpl13a)* in the liver, spleen and bone marrow to show total gene expression. In addition, in the spleen and bone marrow samples, each gene of interest was also normalized to the erythroid specific marker *glycophorin A (GypA)* to show changes in gene expression relative to erythroid cells and to account for any erythroid expansion within the tissues [25]. The validation of primers and analysis is consistent with the MIQE guidelines [26]. All changes in gene expression were expressed relative to wild-type littermates or appropriate control animals, and data are expressed as a fold change compared to the relevant control. Primer pairs for each gene are shown in Table 1.

**Table 1:** Sequences of primers used for qPCR.

Gene name	Primer sequence
*Bmp6*	Forward—AACAGCTTGCAAGAAGCATGAG
	Reverse—TGGACCAAGGTCTGTACAATGG
*Erfe*	Forward—CCAGGCCCCTTTATCCCATC
	Reverse—GTGCTCCAGATGGCTCTCTC
*Gdf15*	Forward—AGCTGTCCGGATACTCAGTCCA
	Reverse—GCTTCAGGGGCCTAGTGATGT
*Gypa*	Forward—GTGATGGCAGGGATTATCGGA
	Reverse—CACTGTTGTCACCACCCTCA
*Hamp1*	Forward—CCTGAGCAGCACCACCTATC
	Reverse—TGCAACAGATACCACACTGGG
*Rpl13a*	Forward—CCATTGTGGCCAAGCAGGTA
	Reverse—TCGGGAGGGGTTGGTATTCA
*Tfr1*	Forward—TCATGAGGGAAATCAATGATCG
	Reverse—CCCCAGAAGATATGTCGGAAAG
*Twsg1*	Forward—GTCTGTTCCCAGCAACAATGTC
	Reverse—TGAAACCAGCGATACTTGGATG

### Quantitation of soluble transferrin receptor 1 (sTfr1)

The ELISA used to measure serum sTfr1 levels was based on a previously published protocol [27]. Rat antimouse CD71 (SouthernBiotech, Birmingham, AL)(100uL; 5μg/mL) in 0.05M carbonate buffer pH 9.1 was added to each well of a 96 well Costar EIA/RIA high binding plate (Sigma Aldrich, Castle Hill, Australia), covered in plastic wrap, and incubated overnight at 4°C. The following morning each well was washed 3 times with wash buffer (phosphate buffered saline containing 0.05% Tween-20). Blocking buffer (5% skim milk powder in wash buffer)(300μL) was then added to each well and the plate sealed with plastic wrap and incubated for one hour. All incubations were carried out at room temperature with constant shaking on an orbital shaker at 150 RPM unless otherwise indicated. The blocking buffer was removed, 100μL of test serum (diluted 1 in 50 in blocking buffer) was added to each well, and the plate was sealed with plastic wrap and incubated for one hour. Each well was then washed three times with wash buffer before 100μL of 0.25μg/mL biotin labelled rat antimouse CD71 (BD, North Ryde, Australia) in blocking buffer was added, the plate sealed with plastic wrap and incubated for one hour. After incubation, the wells were washed three times with wash buffer and 100μL of streptavidin-HRP (1:8000, Cell Signaling Technology, Danvers, MA) in blocking buffer was added. The plate was sealed with plastic wrap and incubated for one hour. Each well was then washed eight times with wash buffer, 100μL of TMB Substrate Solution (Cell Signaling Technology) was added to each well and the plate was incubated for 15 minutes at room temperature in the dark without shaking. Stop Solution (Cell Signalling Technology)(100μL) was then added and the absorbance measured at 450nm using a Synergy H4 Hybrid plate reader (BioTek, Winooski, VT). Relative sTfR1 levels were determined from a standard curve produced using serum from *hbd* mice.

### Statistical analysis

All experiments contained between 4 and 9 mice per group and the values represent mean ± standard error of the mean (SEM). The statistical differences between each group and their respective controls were calculated with Student’s t-test using IBM SPSS Statistics version 22 software (IBM Australia, St Leonards, Australia) and a P value of <0.05 was considered significant.

## Results

### Hematological parameters, serum erythropoietin levels and *Hamp1* expression in mouse anemia models

**Fig 1:**
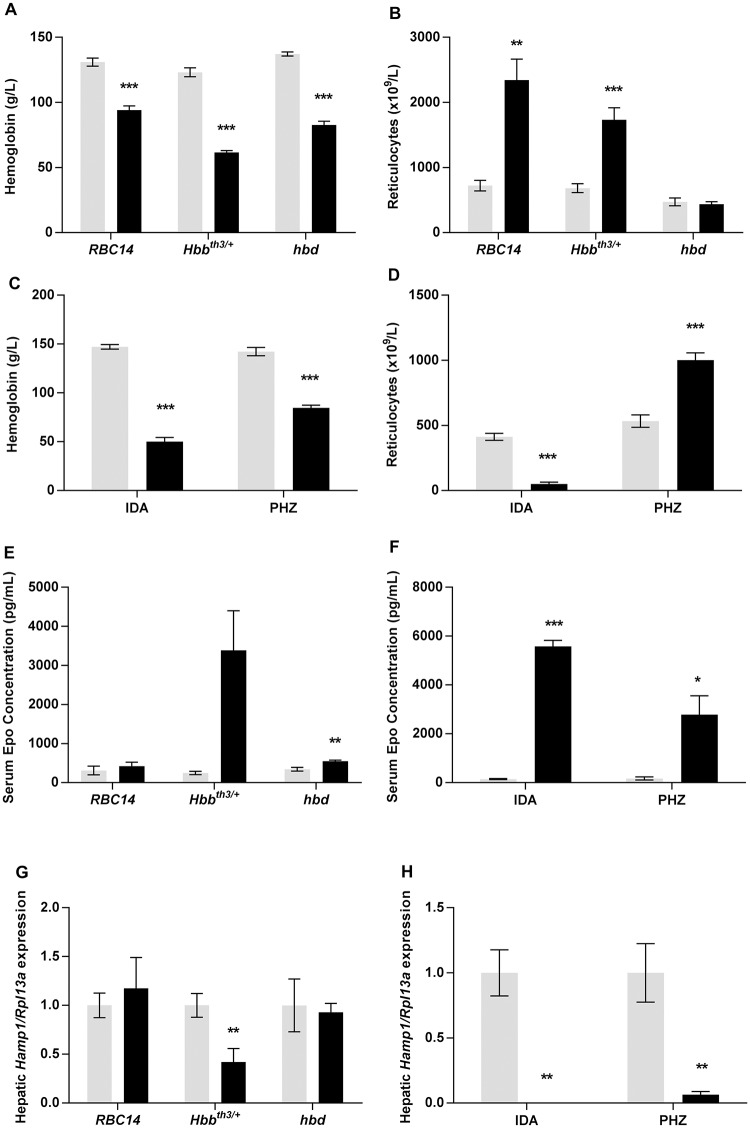
Hematological characteristics and hepatic *Hamp1* expression in mouse models of anemia. Tissues were taken from *RBC14*, *Hbb*^*th3/+*^ and *hbd* mice, and littermate controls for each strain, as well as mice maintained on an iron deficient diet and mice treated with PHZ. Subsequent analyses were carried out as described in the Materials and Methods section. Hemoglobin concentration (A, C), reticulocyte count (B, D), serum erythropoietin levels (E, F) and relative hepatic *Hamp1* expression (G, H) were determined for each mouse model. *Hamp1* expression levels were calculated relative to the housekeeping gene *Rpl13a*, and are expressed as a proportion of the values for wild-type littermates (for *Hbb*^*th3/+*^, *RBC14* and *hbd* mice), mice fed the control diet (for the iron deficient mice) or untreated animals (for the PHZ-injected cohort). The data represent mean ± SEM. Light bars represent control groups and dark bars represent models of anemia. IDA–iron deficient study; PHZ–PHZ treatment study. Significant differences were calculated relative to the respective control groups. *P < 0.05; ** *P* < 0.01; ***P < 0.001.

Analysis of hemoglobin levels showed that all of the mouse models were anemic. Of the genetic models, *Hbb*^*th3/+*^ mice had the lowest hemoglobin (50% of wild-type values) (Fig 1A) with the anemia exhibited by *hbd* mice being less severe (60% of wild-type values). As previously reported [19], *RBC14* mice exhibited a relatively mild anemia with hemoglobin values dropping to 72% of wild-type levels. The anemia was associated with reticulocytosis in *RBC14* and *Hbb*^*th3/+*^ mice (reticulocyte count 3.2-fold and 2.5-fold higher respectively). However, no change in reticulocyte count was seen in the *hbd* strain (Fig 1B) despite obvious anemia. This is likely due to the reduction in transferrin bound iron uptake by *hbd* erythroid precursors, resulting in the inhibition of erythroid cell maturation [28]. The mice maintained on an iron deficient diet were by far the most anemic of the groups studied, with hemoglobin levels 34% of that found in mice fed the control diet, whereas the values for the phenylhydrazine treated mice were 60% of control values (Fig 1C). In contrast to the other groups, reticulocytes were significantly decreased in iron deficient mice to 12% of that seen in mice fed the control diet (Fig 1D), as the maturation of developing erythroblasts is impeded by the severe iron deficiency [29]. Similar to most of the anemic models, reticulocytosis was also evident in phenylhydrazine-treated mice (1.9-fold increase)(Fig 1D). In general, serum erythropoietin levels were inversely related to the severity of the anemia (Fig 1E and 1F). The iron deficient group showed the greatest increase in erythropoietin (40-fold), with the *Hbb*^*th3/+*^ mice having the highest concentration of the genetic anemia strains, although this did not reach statistical significance (P = 0.053). Surprisingly, *RBC14* mice did not show an increase in serum erythropoietin levels, although this could be due to the mild anemia exhibited by these animals.

Interestingly, of the genetic forms of anemia, only *Hbb*^*th3/+*^ mice had reduced hepatic *Hamp1* mRNA expression (42% of wild-type levels) (Fig 1G), despite all mice exhibiting anemia. In contrast, *Hamp1* message levels were drastically decreased in the iron deficient group (>10,000-fold reduction) and the phenylhydrazine treated mice (6% of untreated mice) (Fig 1H). These results demonstrate that a range of *Hamp1* expression responses can be obtained from the different models of anemia, making them useful tools with which to examine the expression of the putative erythroid regulators.

### Expression of putative erythroid regulators

**Fig 2:**
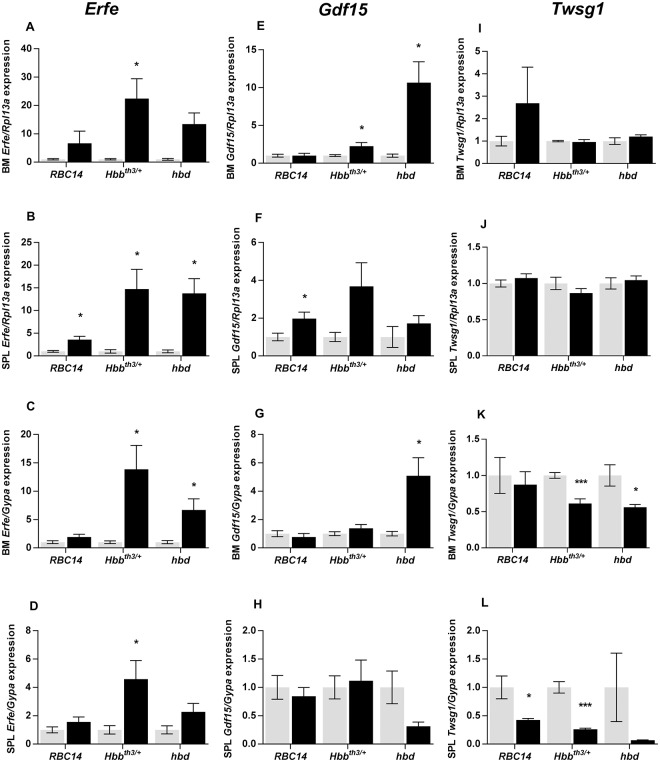
Expression of putative erythroid regulators in genetic model of anemia. Tissues were taken from *RBC14*, *Hbb*^*th3/+*^ and *hbd* mice (and littermate controls) and gene expression in the bone marrow and spleen was determined by qPCR as described in the Materials and Methods section. Relative *Erfe* (A-D), *Gdf15* (E-H) and *Twsg1* (I-L) expression was determined for each model. Gene expression levels were calculated relative to the housekeeping gene *Rpl13a* for total tissue expression or relative to *GypA* for expression in developing erythroid cells, and are expressed as a proportion of wild-type littermates for each strain. The data represent mean ± SEM. Light bars represent control groups and dark bars represent models of anemia. Significant differences were calculated relative to the respective control groups. *P < 0.05; ***P < 0.001.

The expression of the genes encoding erythroferrone, Gdf15 and Twsg1, which have previously been proposed to play a role as erythroid regulators, were studied in the bone marrow and spleen. Significant increases in total *Erfe* mRNA expression in the bone marrow of *Hbb*^*th3/+*^ and *hbd* mice (22-fold and 13-fold respectively) and the spleen of all three genetic anemia strains (*RBC14*–4-fold, *Hbb*^*th3/+*^– 15-fold, *hbd*– 14-fold) were observed (Fig 2A and 2B). When presented as a proportion of *Gypa* expression (which varies in direct proportion to the number of erythroid precursors), bone marrow *Erfe* expression in *Hbb*^*th3/+*^ and *hbd* mice remained elevated, but not to the same extent as that seen for total gene expression (14-fold and 6.7-fold respectively) (Fig 2C). The difference in total and per erythroid precursor gene expression was even more pronounced in the spleen, with increases in *Erfe* expression reduced to 4.6-fold and 2.3-fold in *Hbb*^*th3/+*^ and *hbd* mice respectively (Fig 2D). These differences in expression indicate an expansion of erythroid activity in the tissues examined with greater expansion in the spleen. Changes in total *Gdf15* mRNA levels were less robust than changes in *Erfe*. Increases were seen in the bone marrow in *Hbb*^*th3/+*^ and *hbd* mice (2 and 10-fold respectively), but only *RBC14* mice showed a significant increase in splenic expression (2-fold) (Fig 2E and 2F). When expressed per erythroid cell, increased *Gdf15* expression was seen in *hbd* bone marrow samples only (Fig 2G and 2H). No changes in total *Twsg1* gene expression were observed in any of the genetic models of anemia (Fig 2I and 2J), but interestingly expression levels per erythroid cell decreased in both the bone marrow and spleen of *Hbb*^*th3/+*^ and *hbd* mice (Fig 2K and 2L).

**Fig 3:**
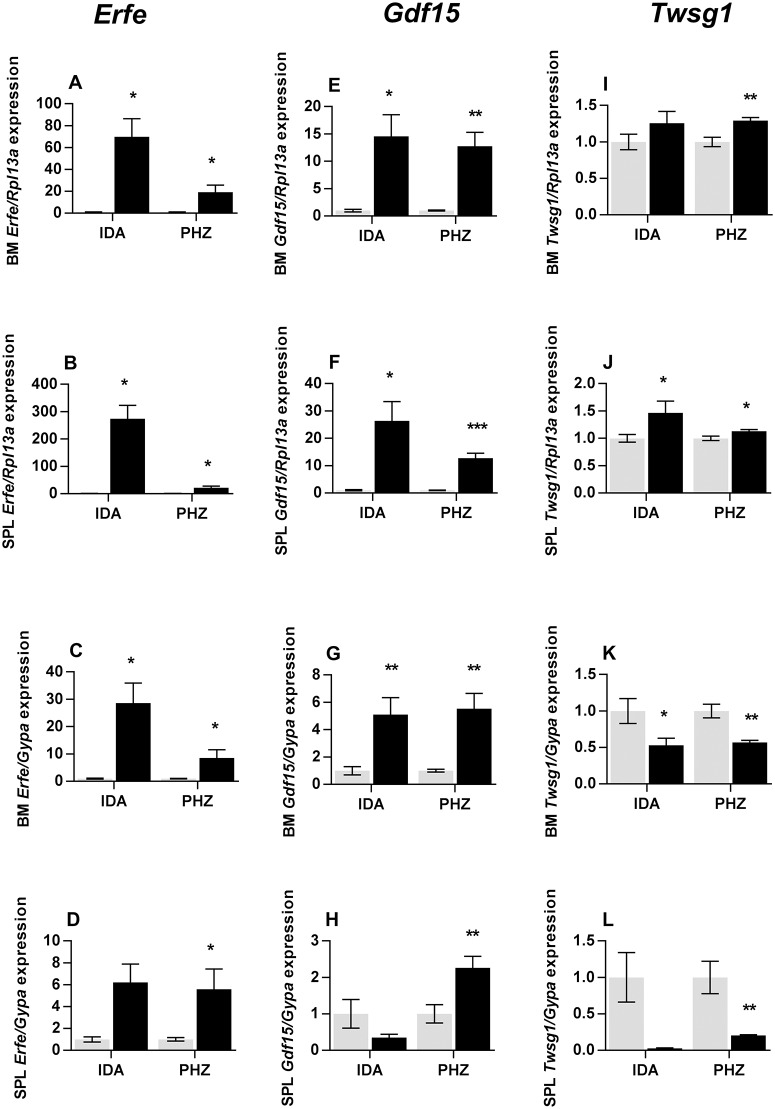
Expression of putative erythroid regulators in iron deficient and PHZ treated mice. Tissues were taken from male mice maintained on an iron deficient diet or treated with PHZ, and gene expression in the bone marrow and spleen were determined by qPCR as described in the Materials and Methods section. Relative *Erfe* (A-D), *Gdf15* (E-H) and *Twsg1* (I-L) expression were determined for each model. Gene expression levels were calculated relative to the housekeeping gene *Rpl13a* for total tissue expression, or relative to *GypA* for expression in developing erythroid cells and are expressed as a proportion of mice fed the control diet for the iron deficient mice or untreated mice for the PHZ-injected cohort. The data represent mean ± SEM. Light bars represent control groups and dark bars represent models of anemia. IDA–iron deficient study; PHZ–PHZ treatment study. Significant differences were calculated relative to the respective control groups. *P < 0.05; ** *P* < 0.01; ***P < 0.001.

A much greater increase in total *Erfe* expression was observed in iron deficient wild-type mice than in any other model examined, with bone marrow and splenic expression increasing 70-fold and 275-fold respectively, compared with mice on the control diet (Fig 3A and 3B). As with the genetic models of anemia, increases in gene expression were not as extensive when expressed per erythroid cell, with *Erfe* levels 29-fold and 8.6-fold higher respectively in bone marrow and spleen (Fig 3C and 3D). *Erfe* mRNA expression also increased in the bone marrow and spleen of phenyhydrazine treated mice (Total: 19-fold and 22-fold respectively, Fig 3A and 3B; Erythroid-normalized: 8.6-fold and 6.6-fold respectively, Fig 3C and 3D) although not to the same extent as in iron deficient mice. Bone marrow and splenic total *Gdf15* message levels were increased in iron deficient mice (15-fold and 26-fold respectively) and phenylhydrazine-treated animals (13-fold for both tissues) (Fig 3E and 3F). However, when the results were expressed on an erythroid cell basis, consistent increases were seen only in phenylhydrazine-treated mice (bone marrow– 5.5-fold, spleen– 2.3-fold) (Fig 3G and 3H). Little change was seen in total *Twsg1* expression in either model, with only modest increases in the spleen of iron deficient (1.5-fold) and phenylhydrazine treated mice (1.1-fold) (Fig 3I and 3J). When expressed on a per cell basis, *Twsg1* decreased in both models (Fig 3K and 3L).

**Fig 4:**
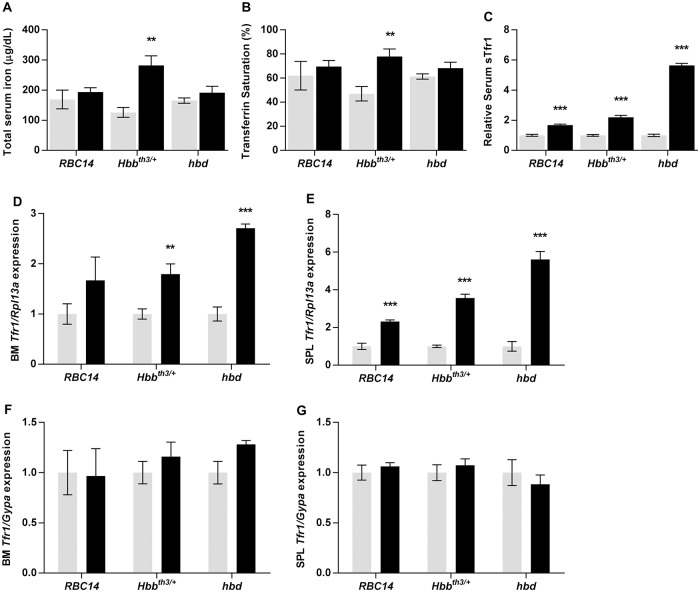
Serum iron parameters and *Tfr1* expression levels in genetic models of anemia. Tissues were taken from *Hbb*^*th3/+*^, *RBC14* and *hbd* mice, and their littermate controls for each strain, and subsequent analyses were carried out as described in the Materials and Methods section. Serum iron concentration (A), transferrin saturation (B), serum sTfr1 levels (C) and relative *Tfr1* gene expression levels (D-G) were determined for each model. Gene expression levels were expressed relative to the housekeeping gene *Rpl13a* for total tissue expression, or relative to *GypA* for expression in developing erythroid cells, and shown as a proportion of the wild-type littermate values for each strain. The data represent mean ± SEM. Light bars represent control groups and dark bars represent models of anemia. Significant differences were calculated relative to the respective control groups. ** *P* < 0.01; ***P < 0.001.

**Fig 5:**
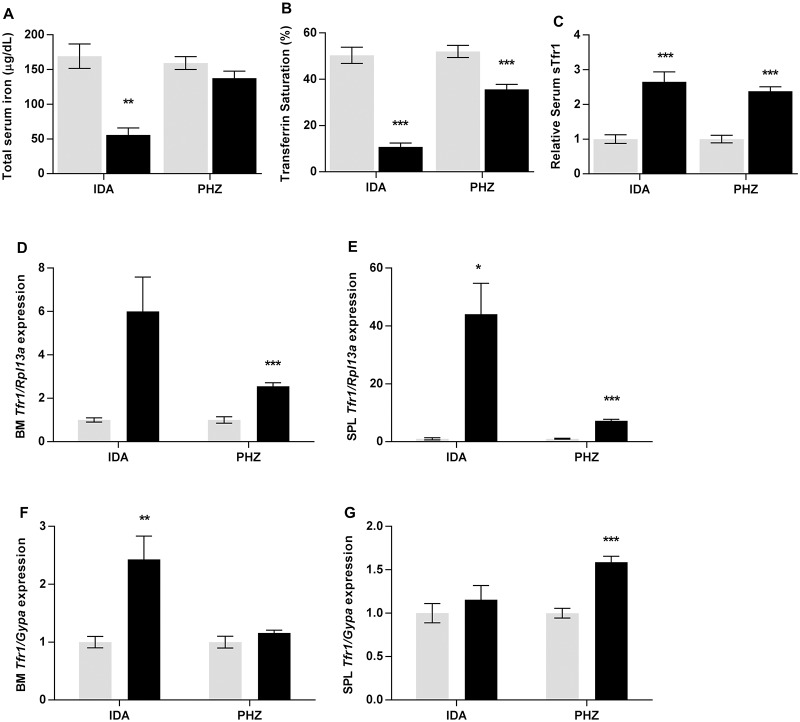
Serum iron parameters and *Tfr1* expression levels in iron deficient and PHZ treated mice. Tissues were taken from male mice maintained on an iron deficient diet or treated with PHZ, and subsequent analyses were carried out as described in the Materials and Methods section. Serum iron concentration (A), transferrin saturation (B), serum sTfr1 levels (C) and relative Tfr1 gene expression levels (D-G) were determined for each model. Gene expression levels were calculated relative to the housekeeping gene *Rpl13a* for total tissue expression, or relative to *GypA* for expression in developing erythroid cells and are expressed as a proportion of mice fed the control diet for the iron deficient mice, or untreated mice for the PHZ-injected cohort. The data represent mean ± SEM. Light bars represent control groups and dark bars represent models of anemia. IDA–iron deficient study; PHZ–PHZ treatment study. Significant differences were calculated relative to the respective control groups. *P < 0.05; ** *P* < 0.01; ***P < 0.001.

The results above show that, while stimulated erythropoiesis leads to an increase in *Erfe* and *Gdf15* expression, much higher levels are reached when the iron supply to erythroid precursors is limited, as in iron deficiency. To investigate the iron available to erythroid precursors, we examined serum iron levels and transferrin saturation. Of the genetic models of anemia, serum iron concentration and transferrin saturation were elevated in *Hbb*^*th3/+*^ mice as has been previously reported [19], but no changes were evident in *RBC14* or *hbd* mice (Fig 4A and 4B). However, normal or even elevated iron in the circulation is, in itself, no guarantee that the supply to erythroid cells is adequate. For example, it has been suggested that iron supply can be limiting in conditions such as β-thalassemia due to the greatly expanded iron requirements of the erythroid compartment, despite a normal or elevated serum iron concentration [30, 31]. To investigate this, we measured serum levels of soluble transferrin receptor 1 (sTfr1), a clinical marker of both stimulated erythropoiesis and functional iron deficiency [32]. sTfr1 was increased in all genetic models (Fig 5C), with levels highest in *hbd* mice (5.6-fold), as has been reported previously [33]. We also examined *Tfr1* mRNA levels in bone marrow and spleen as the level of this transcript is increased when intracellular iron is limiting. *Tfr1* message was increased in the bone marrow of each of the three genetic models of anemia, but only reached statistical significance for *Hbb*^*th3/+*^ and *hbd* mice (1.8-fold and 2.7-fold increases respectively). Increased Tfr1 mRNA was observed in the spleen of all three genetic models of anemia (*RBC14*–2.3-fold, *Hbb*^*th3/+*^– 3.6-fold, *hbd*– 5.6-fold) (Fig 4D and 4E). Interestingly, no change in the expression of *Tfr1* message per erythroid cell was detected in any of the genetic models (Fig 4F and 4G), implying that the iron supply to developing red blood cells is not limiting in these situations.

As expected, both serum iron levels and transferrin saturation were significantly decreased in iron deficient mice (33% and 22% of controls respectively) (Fig 5A and 5B). Transferrin saturation was also decreased to 68% of control levels in phenylhydrazine-treated mice (Fig 5B), possibly limiting the iron available to erythroid progenitor cells in this model as well. The level of sTfr1 was increased approximately 2.5-fold in both models, which was similar to that seen in *Hbb*^*th3/+*^ mice, and less than half that of *hbd* mice (Fig 5C). When tissue *Tfr1* mRNA expression was examined, the largest increases were seen in the bone marrow and spleen of iron deficient mice (6-fold and 44-fold respectively) although the increase in bone marrow did not reach statistical significance (p = 0.051) (Fig 5D and 5E). *Tfr1* mRNA levels were also significantly elevated in both the bone marrow and spleen of phenylhydrazine-treated mice (2.6-fold and 7.2-fold respectively) (Fig 5D and 5E). The expression of *Tfr1* message per erythroid cell was more than doubled in iron deficient bone marrow, whereas no change was seen in the spleen of these mice. The lack of increase in splenic expression is surprising given that iron supply is almost certainly compromised in iron deficient mice and suggests that *Tfr1* expression is already at its maximum in splenic erythroid progenitors. However, an increase in *Tfr1* expression per erythroid cell was seen in the spleen of phenylhydrazine treated mice (1.6-fold)(Fig 5G). The reason for this is unclear, although it may reflect temporal differences rather than actual cellular iron status, with phenylhydrazine treatment causing a very rapid, acute expansion of erythroid cells as opposed to the chronic increase in erythropoietic drive experienced in the other models.

### Contribution of the stores regulator to *Hamp1* expression in mouse models of anemia

**Fig 6:**
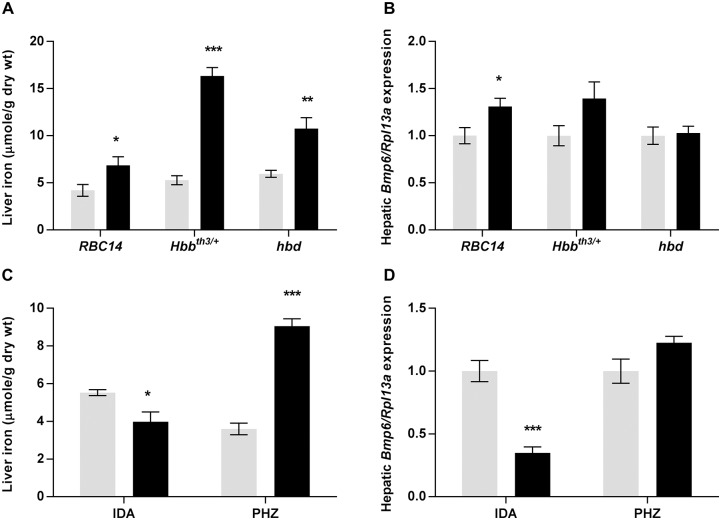
Liver iron concentration and hepatic *Bmp6* expression in mouse models of anemia. Tissues were taken from *Hbb*^*th3/+*^, *RBC14* and *hbd* mice, and littermate controls, mice maintained on an iron deficient diet and mice treated with PHZ, and subsequent analyses were carried out as described in the Materials and Methods section. Liver iron concentration (A, C) and relative hepatic *Bmp6* expression (B, D) were determined for each mouse model. *Bmp6* expression was calculated relative to the housekeeping gene *Rpl13a* and is shown as a proportion of relevant control values (see Materials and Methods). The data represent mean ± SEM. Light bars represent control groups and dark bars represent models of anemia. IDA–iron deficient study; PHZ–PHZ treatment study. Significant differences were calculated relative to the respective control groups. *P < 0.05; ** *P* < 0.01; ***P < 0.001.

The regulation of *Hamp1* expression during erythropoiesis is not solely under the control of the erythroid regulator, with the stores regulator also playing a role, particularly in the iron loading anemias. Thus, we have examined the likely contribution of the stores regulator to hepcidin production in our anemic mouse models. Hepatic iron stores were elevated in each of the genetic models of anemia studied, with *Hbb*^*th3/+*^ accumulating the most iron (3-fold more than wild-type littermates) (Fig 6A). However, only minor changes in hepatic *Bmp6* expression were detected (Fig 6B), suggesting that, at this age, the stores regulator has little effect on *Hamp1* expression in these animals. In contrast, the reduction in iron stores in the iron deficient group (Fig 6C) resulted in hepatic *Bmp6* expression decreasing to 35% of control levels (Fig 6D). This makes it likely that the combined influence of the erythroid regulator and the stores regulator causes the extremely low *Hamp1* expression seen in these mice. As expected, iron stores were increased in phenylhydrazine treated animals (2.5-fold) (Fig 6C), however, like *Hbb*^*th3/+*^ mice, *Bmp6* expression remained unchanged (Fig 6D). The lack of *Bmp6* up-regulation in these models despite increases in liver iron concentration is likely due to the location of the iron within the liver, as non-parenchymal iron does not induce *Bmp6* expression [34], and the iron in both young *Hbb*^*th3/+*^ mice and phenylhydrazine injected mice is likely to localise predominantly to Kupffer cells. Overall, these results suggest that the contribution of the stores regulator to *Hamp1* regulation is negligible in all models examined, apart from the iron deficient group.

## Discussion

Under normal conditions, both the stores regulator and the erythroid regulator act via hepcidin to ensure an adequate supply of iron to body cells, while minimising the accumulation of excess iron [1]. The stores regulator responds to changes in body iron levels, and specifically liver iron levels, and signals changes in hepcidin via the BMP6/SMAD pathway. The erythroid regulator acts to maintain iron supply specifically to developing red blood cells, as these cells are quantitatively the most important users of iron in the body, taking up more than 80% of the iron entering the circulation [7]. The proteins GDF15, TWSG1 and, more recently, erythroferrone, have all been proposed to signal the iron requirements of erythroid tissue to the liver [9, 10, 13]. In this study, we have examined the expression of these putative erythroid regulators in a range of mouse models of anemia. We have measured gene expression using both a housekeeping gene that is ubiquitously expressed, and a second that is expressed only erythroid cells. This strategy has allowed us to take into account expansion of the erythroid tissue that occurs in models such as *Hbb*^*th3/+*^ mice and phenylhydrazine treated animals. In the absence of reliable commercial reagents for measuring serum levels of the putative erythroid regulators, it is not possible to comment definitively on the relative effects of the regulators on hepcidin expression, so we have limited our discussion to the effect of various erythropoietic stimuli on the expression of erythroid candidate genes.

Of the three candidate erythroid regulators, erythroferrone is by far the most promising. *Erfe* expression is upregulated in *Hbb*^*th3/+*^ mice, and following erythropoietin treatment and phlebotomy, all of which are associated with a reduction in *Hamp1* expression. Importantly, *Erfe* knockout mice fail to reduce *Hamp1* expression to the same extent as wild-type mice following phlebotomy [13]. In addition, *Erfe* knockout mice on a *Hbb*^*th3/+*^ background have normal *Hamp1* expression and do not accumulate as much iron as *Hbb*^*th3/+*^ animals alone. In the current study, we found that *Erfe* was the most consistently upregulated of the three candidate erythroid regulators in response to anemia. *Erfe* gene expression was also far more highly expressed in iron deficiency than in any other model tested. This makes it an ideal erythroid candidate. In the original paper outlining the concept of the stores and erythroid regulators [7], Finch stated that the erythroid regulator was not a function of the rate of erythropoiesis *per se*, but was instead related to the adequacy of iron supply to the marrow. He argued that it was possible to increase erythropoiesis several fold without altering iron absorption (hepcidin had not yet been discovered) as long as the iron supply to erythroid precursors was not limiting. Therefore, the expression of an erythroid regulator should be maximal during severe iron deficiency, when iron supply is at its most limiting.

Although the increase in *Erfe* expression was greater in both the bone marrow and spleen of iron deficient mice than in any of the other models examined, the difference between strains reduced significantly when expression was normalized for the amount of active erythroid tissue (based on glycophorin A expression), particularly in the spleen. This indicates that much of the increase in splenic *Erfe* expression in iron deficiency is due to an increase in the number of erythroid precursors, rather than an increase in the expression of *Erfe* per cell. This is likely due to the increase in circulating erythropoietin stimulating erythroid expansion, as erythropoietin levels were highest in the iron deficient model. In contrast, in the bone marrow the increase in *Erfe* is predominantly due to an increase in per erythroid cell expression. This may be related to intracellular iron deficiency as bone marrow erythroid precursors expressed higher levels of *Tfr1* mRNA in this model, or it may represent a direct effect of higher levels of erythropoietin on *Erfe* expression. This tissue specific relationship between total and erythroid cell specific *Erfe* expression was also seen in most of the other models examined in this study and agrees with the *Erfe* expression pattern reported recently in *Hbb*^*th3/+*^ mice [35].

Of the other candidate molecules, GDF15 is the most likely to be involved in hepcidin regulation. It remains the only candidate erythroid regulator to be detected in human serum and is elevated in many conditions associated with stimulated erythropoiesis [36–38]. In our study, *Gdf15* expression was upregulated in the bone marrow and spleen of iron deficient and phenylhydrazine treated mice, however, unlike *Erfe* expression, no major differences between the two models was observed. Others have failed to find a link between circulating GDF15 levels and hepcidin production in chronic kidney disease [39], pregnancy [40] and various red cell disorders in humans [41]. An alternative role for GDF15 was suggested by Ramirez *et al*. who demonstrated that the cytokine is required for the differentiation of human erythroid cells in culture [37], implying that circulating GDF15 levels are a by-product of erythropoiesis rather than a signal to reduce hepcidin. However, no inhibition of erythroid maturation was observed in phlebotomised *Gdf15* knockout mice [11], so the role of GDF15 remains uncertain.

In contrast to *Erfe* and *Gdf15*, *Twsg1* expression remained largely unchanged in the bone marrow and spleen, with expression per erythroid cell decreasing in most models examined. This agrees with several recent studies which show no change in *Twsg1* expression in the spleen and bone marrow of phlebotomized wild-type mice [11] and *Hbb*^*th3/+*^ mice [35], although other, earlier studies reported an increase in splenic *Twsg1* expression in *Hbb*^*th3/+*^ mice [10, 42]. While the reason for this difference is unclear, the earlier studies examined gene expression in much older animals (8–12 weeks) than we used, and the expression of many iron related genes, including *Hamp1*, is altered with age [35]. While studies showing a lack of *Tswg1* gene regulation during stimulated erythropoiesis do not rule out a role for this protein in hepcidin regulation, the current evidence suggests that TWSG1 is unlikely to act as an erythroid regulator under most circumstances.

A potential role for sTFR1 in regulating hepcidin production in response to an erythropoietic stimulus has also been suggested previously [43]. This serum protein is widely used clinically as a marker of functional iron deficiency [32], making it an ideal candidate for an erythroid regulator. However, a previous study in mice using the hydrodynamic injection technique to increase circulating sTfr1 levels failed to record any change in *Hamp1* expression, although the increase in mouse sTfr1 in the circulation was not directly confirmed [44]. In addition, hydrodynamic injections can have numerous effects on the liver [45], which could potentially disrupt hepatic *Hamp1* regulatory pathways. In the current study, we show that *hbd* mice naturally overproduce sTfr1 to levels double that seen in the circulation of iron deficient or *Hbb*^*th3/+*^ mice, providing an ideal model with which to examine the effect of elevated sTfr1 levels on *Hamp1* expression. As we saw no change in *Hamp1* expression in *hbd* mice and reductions in *Hamp1* expression in models with lower levels of circulating sTfr1, our findings suggest that sTfr1 does not play a role in *Hamp1* regulation.

In conclusion, this study supports the prevailing hypothesis that erythroferrone is the most promising of the erythroid candidates proposed to date, whereas GDF15 and TWSG1 are likely to have little or no effect on hepcidin production. *Erfe* gene expression is stimulated most highly when iron supply to developing erythroid cells is limiting, particularly in the spleen, although this is associated predominantly with the expansion of the erythroid compartment. When appropriate tools become available, further studies examining serum levels of candidate erythroid regulators are required to fully characterise their role in the regulation of hepcidin expression.

## Supporting Information

S1 File Raw Data. (XLSX)
